# Chlorogenic Acid Induced Neuroblastoma Cells Differentiation via the ACAT1-TPK1-PDH Pathway

**DOI:** 10.3390/ph16060877

**Published:** 2023-06-14

**Authors:** Shen You, Ming-Jin Wang, Zhen-Yan Hou, Wei-Da Wang, Ting-Ting Du, Ni-Na Xue, Ming Ji, Xiao-Guang Chen

**Affiliations:** 1 State Key Laboratory of Bioactive Substances and Functions of Natural Medicines, Institute of Materia Medica, Chinese Academy of Medical Sciences and Peking Union Medical College, Beijing 100050, China; youshen@imm.ac.cn (S.Y.); wangmingjin@imm.ac.cn (M.-J.W.); houzhenyan@imm.ac.cn (Z.-Y.H.); wangweida@imm.ac.cn (W.-D.W.); ninadu@imm.ac.cn (T.-T.D.); angelnina@imm.ac.cn (N.-N.X.); 2 Beijing Key Laboratory of New Drug Mechanisms and Pharmacological Evaluation Study, Institute of Materia Medica, Chinese Academy of Medical Sciences and Peking Union Medical College, Beijing 100050, China; 3 Key Laboratory of Small Molecule Immuno-Oncology Drug Discovery, Chinese Academy of Medical Sciences, Beijing 100050, China

**Keywords:** neuroblastoma, chlorogenic acid, differentiation, acetyl-CoA acetyltransferase, thiamine

## Abstract

Background: Chlorogenic acid (CHA) has been shown to have substantial biological activities, including anti-inflammatory, antioxidant, and antitumor effects. However, the pharmacological role of CHA in neuroblastoma has not yet been assessed. Neuroblastoma is a type of cancer that develops in undifferentiated sympathetic ganglion cells. This study aims to assess the antitumor activity of CHA against neuroblastoma and reveal its mechanism of action in cell differentiation. Methods: Be(2)-M17 and SH-SY5Y neuroblastoma cells were used to confirm the differentiation phenotype. Subcutaneous and orthotopic xenograft mouse models were also used to evaluate the antitumor activity of CHA. Seahorse assays and metabolomic analyses were further performed to investigate the roles of CHA and its target ACAT1 in mitochondrial metabolism. Results: CHA induced the differentiation of Be(2)-M17 and SH-SY5Y neuroblastoma cells in vivo and in vitro. The knockdown of mitochondrial ACAT1, which was inhibited by CHA, also resulted in differentiation characteristics in vivo and in vitro. A metabolomic analysis revealed that thiamine metabolism was involved in the differentiation of neuroblastoma cells. Conclusions: These results provide evidence that CHA shows good antitumor activity against neuroblastoma via the induction of differentiation, by which the ACAT1-TPK1-PDH pathway is involved. CHA is a potential drug candidate for neuroblastoma therapy.

## 1. Introduction

Neuroblastoma has a high incidence in children and is a solid tumor of the extracranial nervous system. It originates from the embryonic neural crest and accounts for 8% of childhood malignancies [1]. Currently, the clinical therapies available for high-risk groups are multi-modality therapies, including chemotherapy, stem cell transplantation, surgery, radiotherapy, tretinoin therapy, and immunotherapy. However, none of these inhibitors can inhibit tumor recurrence or metastasis [2,3]. Therefore, further research on the mechanisms and treatment options for neuroblastoma is urgently needed.

The relationship between cancer and the stagnation of differentiation has been reported for several years. Neuroblastoma is a cancer that develops from undifferentiated sympathetic ganglion cells. Thus, differentiation-inducing therapy may be possible in this cancer treatment [4]. To date, retinoic acid (RA) is the most effective drug for inducing neuroblastoma differentiation. Neuroblastoma cell lines treated with RA showed an irreversible decrease in proliferative capacity and increased neuroprotrusion formation in neuroblastoma cell lines [5]. The RA derivative 13-cis RA has rapidly entered clinical trials in neuroblastoma and has now become a part of the standard treatment [6]. The basic principle is that 13-cis RA induces residual tumor cell differentiation and reduces the risk of recurrence after chemotherapy, surgical excision, and bone marrow transplantation in patients. However, the mechanism has not been fully elucidated [7]. MYCN amplification (MNA) has been reported in approximately 25% of patients diagnosed with neuroblastoma, and MYCN amplification affected cell differentiation procedures. RA was not an effective treatment option for neuroblastoma patients with MYCN amplification because MYCN amplification caused RA treatment resistance [8,9,10]. Because the molecular mechanisms of neuroblastoma proliferation, differentiation, and survival remain largely unexplored, it would be of clinical benefit to study new differentiation-inducing agents and mechanisms.

Chlorogenic acid (CHA) is a small, natural molecule extracted from foods and plants, such as *Eucommia ulmoides* and coffee. It is a polyhydroxyphenol-based phenyl compound that has been reported to regulate the immune tumor microenvironment and repolarize macrophages from the M2 type to the M1 type in order to inhibit tumor growth [11]. CHA has also been reported to relieve lupus-erythematosus-like skin lesions and arthritis by reducing IL17 [12]. In addition to its effect on immunotherapy, studies have shown that CHA has a good differentiation induction effect in liver cancer and lung cancer, but its role in neuroblastoma has not been reported [13]. In addition, previous studies in our laboratory revealed that CHA could inhibit the activity of Acetyl-CoA acetyltransferase (ACAT1), which is specifically expressed in mitochondria. ACAT1 is a key enzyme in ketone body metabolism and fatty acid metabolism [14]. It has been reported that ACAT1 exists in the form of a tetramer, in which the Y407 site of ACAT1 can make the tetramer more stable and enhance its activity while damaging the stability of the ACAT1 tetramer, and the knockdown of ACAT1 can effectively control tumor growth [15].

Herein, we report a novel mechanism for CHA in neuroblastoma. CHA induces the differentiation of neuroblastoma cells in vitro and in vivo by inhibiting ACAT1. Treatment with CHA or abolishment of ACAT1 restored the oxidative phosphorylation (OXPHOS) of neuroblastoma cells. We found that CHA upregulated the expression of thiamin pyrophosphokinase 1(TPK1), which activates the thiamine metabolic pathway, enhances pyruvate dehydrogenase (PDH) activity, and promotes neuroblastoma cell differentiation.

## 2. Results

### 2.1. CHA-Induced Neuroblastoma Cell Differentiation In Vitro and In Vivo

**Figure 1:**
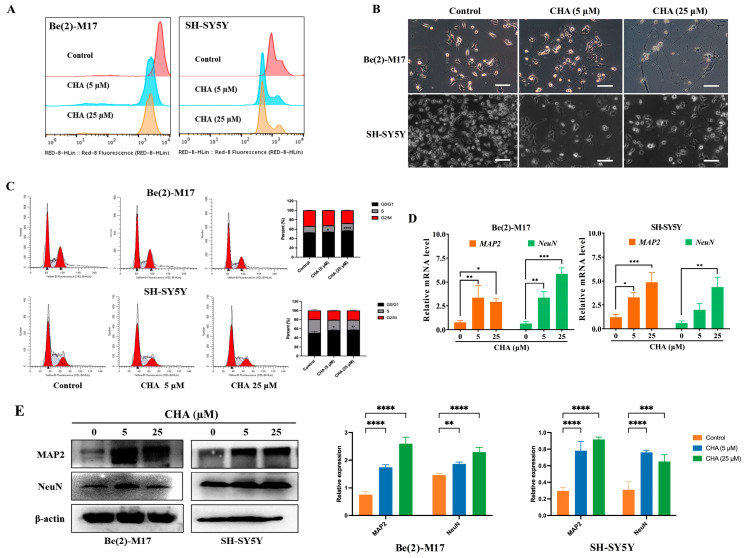
CHA promoted the differentiation of Be(2)-M17 and SH-SY5Y cells. (**A**) The effects of CHA on the cell morphology of Be(2)-M17 and SH-SY5Y cells’ microscopy images were captured. Scale bar: 100 μm. (**B**) EdU fluorescence intensity was determined quantitatively by flow cytometry. (**C**) CHA blocked the cell cycle in the G0/G1 phase. (**D**) The relative mRNA levels of MAP2 and NeuN in two cell lines treated with CHA (5 μM and 25 μM) for 24 h were detected by real-time qPCR, *n* = 3. (**E**) A western blot analysis of MAP2 and NeuN in two cell lines treated with CHA (5 μM and 25 μM) for 24 h, *n* = 3. For (**C**–**E**), * *p* < 0.05, ** *p* < 0.01, *** *p* < 0.001, and **** *p* < 0.0001 compared to the control.

CHA has been clinically evaluated in patients with recurrent high-grade glioma and has exhibited preliminary efficacy [16]. CHA may exhibit antitumor activity against neuroblastoma. Therefore, we first investigated the effect of CHA on neuroblastoma cells. After treatment with CHA for a week, the 5-ethynyl-2′-deoxyuridine (EdU) assay showed that both cell lines exposed to CHA proliferated slowly compared to the control cells (Figure 1A). Surprisingly, two neuroblastoma cells (Be(2)-M17 and SH-SY5Y) displayed obvious morphological changes, including longer neurites and more branches, along with signs of mature cells (Figure 1B). The cell cycle was arrested at the G0/G1 phase after CHA treatment for 24 h (Figure 1C). The abilities of migration and clone formation of both cell types were weaker than those of the control group (Figure S1A,B). However, JC-1 staining, a probe for detecting mitochondrial membrane potential, showed no change after CHA stimulation, neglecting the cytotoxicity of CHA (Figure S1C). Intriguingly, the differentiation markers (MAP2 and NeuN) were upregulated at both mRNA and protein levels in Be(2)-M17 and SH-SY5Y cells in the presence of CHA (Figure 1D,E).

**Figure 2:**
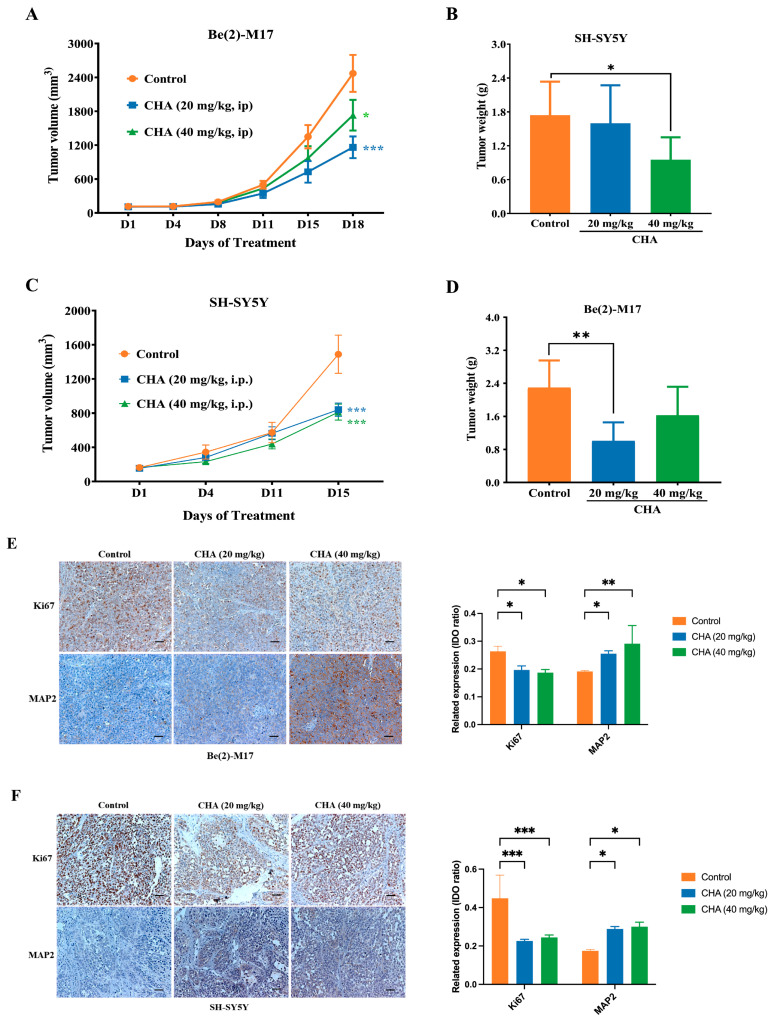
CHA induced the differentiation of Be(2)-M17 and SH-SY5Y models in vivo. (**A**,**B**) Tumor volumes and tumor weights in the Be(2)-M17 model, mice were administrated with CHA (20 mg/kg and 40 mg/kg, i.p.) for 18 days, *n* = 7. (**C**,**D**) Tumor volumes and tumor weights in the Be(2)-M17 model; CHA (20 mg/kg and 40 mg/kg, i.p.) was administrated in mice for 16 days, *n* = 6. (**E**,**F**) The expressions of Ki67 and MAP2 were analyzed by immunohistochemistry, *n* = 3. Scale bar: 100 μm. For (**A**–**F**), * *p* < 0.05, ** *p* < 0.01, *** *p* < 0.001, * represented compared to the control.

To confirm these observations, we evaluated the antitumor activity of CHA in vivo. In mouse xenograft models, CHA showed good antitumor activity against Be(2)-M17 and SH-SY5Y cells (Figure 2A–D). CHA significantly repressed tumor growth without causing a loss of body weight (Figure S2A,B). The mouse orthotopic model also displayed similar results (Figure S2C). The growth of tumors was delayed by a reduction in Ki-67 expression and an elevated level of the MAP2differentiation marker in the CHA group compared to that in the control group (Figure 2E,F). Taken together, CHA induces neuroblastoma cell differentiation.

### 2.2. Mitochondrial ACAT1 Involved in Neuroblastoma Cell Differentiation Induced by CHA

**Figure 3:**
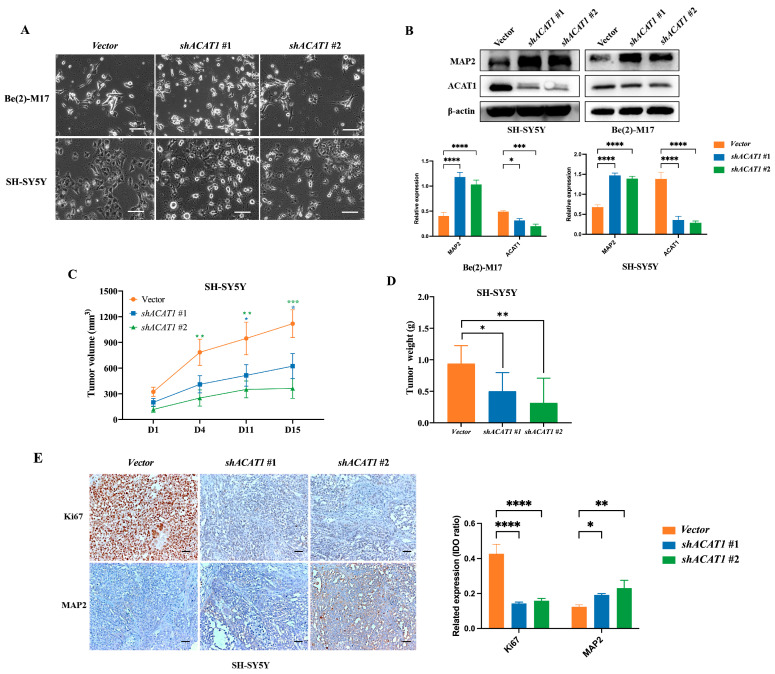
The knockdown of ACAT1 promoted the differentiation of Be(2)-M17 and SH-SY5Y cells in vitro and in vivo. (**A**) Cell morphology of Be(2)-M17 and SH-SY5Y cells in the absence of ACAT1. Microscopy images were captured. Scale bar: 100 μm. (**B**) Western blot analysis of differentiation markers in ACAT1 knockdown cells. β-actin is used as a control, *n* = 3. (**C**,**D**) Tumor volumes and tumor weights in the SH-SY5Y model with ACAT1 knockdown for 16 days, *n* = 7. (**E**) The expressions of Ki67 and MAP2 were analyzed by immunohistochemistry, *n* = 3. Scale bar: 100 μm. For (**B**–**E**), * *p* < 0.05, ** *p* < 0.01, *** *p* < 0.001 and **** *p* < 0.0001, compared to the vector group.

In our previous study, we found that CHA is an inhibitor of mitochondrial ACAT1 (Figure S3A). We conjectured that mitochondrial ACAT1 is involved in the differentiation of neuroblastoma cells induced by CHA. Thus, mitochondrial ACAT1 was knocked down in both cell types. Cell morphology was similar to that of cells treated with CHA, where the cells tended to mature (Figure 3A). Silencing ACAT1 delayed cell proliferation without cytotoxicity (Figure S3B,C) and increased the expression of MAP2 in cells (Figure 3B). Consistently, tumors grew slowly after ACAT1 knockdown (Figure 3C,D). The expression of Ki-67 was reduced, and that of MAP2 was highly upregulated in tumor tissues (Figure 3E). These data implied that mitochondrial ACAT1 is involved in neuroblastoma cell differentiation.

### 2.3. CHA Restored OXPHOS Capacity in Neuroblastoma Cells

**Figure 4:**
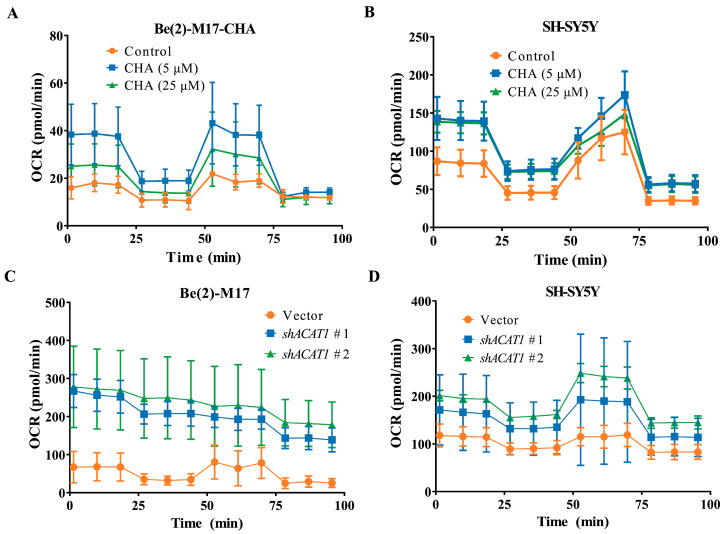
CHA treatment and knockdown of ACAT1 in Be(2)-M17 and SH-SY5Y cells restored OXPHOS. (**A**,**B**) The OCR of Be(2)-M17 and SH-SY5Y cells with the CHA treatment was monitored in real-time using a Seahorse Bioscience Extracellular Flux Analyzer, *n* = 3. (**C**,**D**) The OCR of Be(2)-M17 and SH-SY5Y cells with ACAT1 knockdown was monitored in real time using a Seahorse Bioscience Extracellular Flux Analyzer, *n* = 3.

Previous studies have reported that ACAT1 is specifically expressed in mitochondria and is involved in energy metabolism [14,15]. We then detected the characteristics of mitochondrial metabolism in CHA-treated cells with CHA treatment using the seahorse mitochondrial stress test. As shown in Figure 4A,B, the oxygen consumption rate (OCR) values were significantly higher in both cells treated with CHA. These results were also observed in ACAT1 knockdown (Figure 4C,D). These data indicated that CHA recovered the level of OXPHOS in neuroblastoma cells via the inhibition of ACAT1.

### 2.4. Thiamine Metabolism Involved in the ACAT-Mediated Differentiation of Neuroblastoma Cells

**Figure 5:**
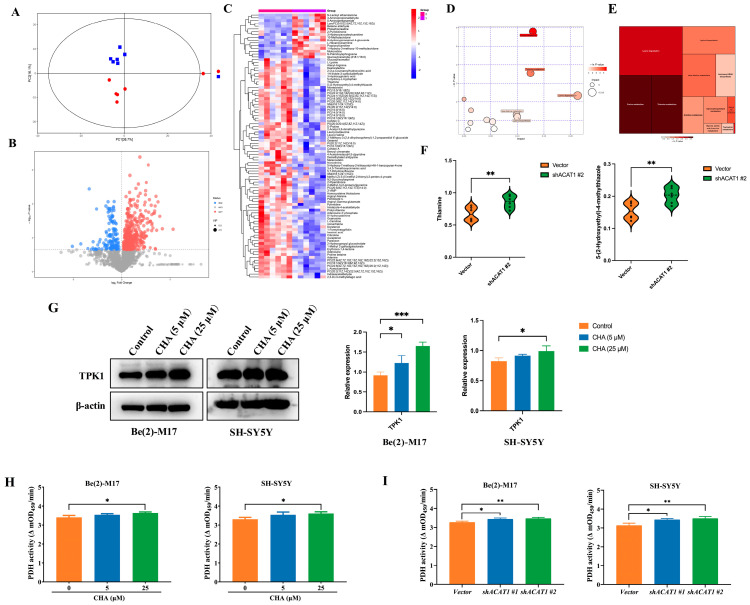
Knockdown of ACAT1 activates thiamine metabolism and enhances PDH activity. (**A**) The PCA analysis of Be(2)-M17 with ACAT1 knockdown, *n* = 6. (**B**) Volcanic maps comparing differential metabolites, *n* = 6. (**C**) Heat map analysis, *n* = 6. (**D**,**E**) The results of metabolic pathway analysis are presented in bubble diagrams and rectangular tree diagrams, respectively. (**F**) Differential metabolites in the thiamine metabolic pathway of cells with ACAT1 knockdown, *n* = 6. (**G**) The expression of TPK1 increased after CHA treatment, *n* = 3. (**H**,**I**) PDH activities. Be(2)-M17 and SH-SY5Y cells with CHA (5 μM and 25 μM) treatment or ACAT1 knockdown, *n* = 3. For (**F**–**I**), * *p* < 0.05, ** *p* < 0.01 and *** *p* < 0.001 compared to the control group.

To explore how ACAT1 affects the differentiation of neuroblastoma cells by regulating mitochondrial metabolism, we performed a metabolomic analysis of Be(2)-M17 cells in the absence of ACAT1, which produced the most remarkable differentiation phenotype. Data were analyzed using principal component analysis (PCA) and partial least squares discriminant analysis (PLS-DA), and then differential metabolites were screened according to *t*-test and variable importance in projection (VIP) values. *p* < 0.05 and differential metabolites with VIP > 1.0 satisfied the adoption criteria. Compared to the vector group, 94 differential metabolites were found in shACAT1 #2 under the positive ion mode, and PCA revealed significant differences between the two groups of principal components (Figure 5A,B). 

KEGG enrichment analysis of these differential metabolites showed that purine metabolism and thiamine metabolism pathways were significantly activated, among which thiamine metabolism is involved in the oxidative decarboxylation of pyruvate and is related to cellular OXPHOS, and thiamine itself has an anti-neuroinflammation effect [16,17] (Figure 5C–E). Compared to the vector, thiamine was significantly upregulated after ACAT1 knockdown, as was the precursor metabolite of thiamine production, 5-hydroxyethyl-4-methylthiazole (Figure 5F). Consistent with the effect of ACAT1 knockdown, CHA also upreglulated the expression of TPK1, a key enzyme that catalyzes the conversion of thiamine to thiamine pyrophosphate (TPP), thereby activating thiamine metabolism (Figure 5G). As thiamine is an important cofactor for PDH, we detected the activity of PDH in both neuroblastoma cell lines. As shown in Figure 5H, CHA enhanced PDH enzymatic activity. In the absence of ACAT1, the activity of PDH also increased compared with that of the vector (Figure 5I).

### 2.5. TPK1 Promoted the Differentiation of Neuroblastoma Cells

**Figure 6:**
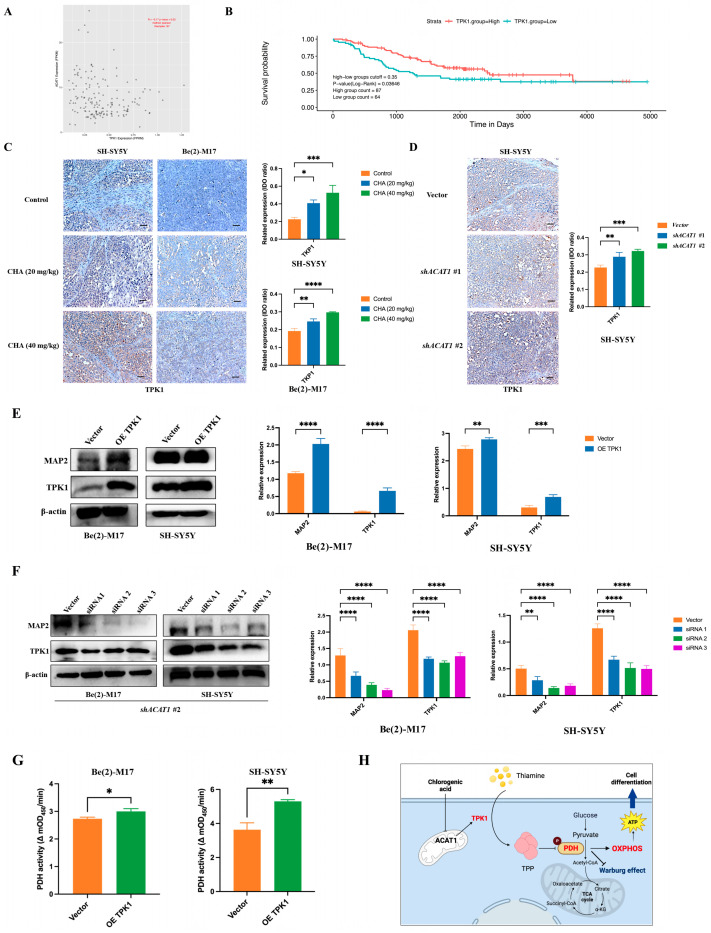
TPK1 promotes Be(2)-M17 and SH-SY5Y cell differentiation. (**A**) The PCAT database showed that ACAT1 was negatively correlated with TPK1. (**B**) Kaplan–Meier curves of overall survival in neuroblastoma patients with different TPK1 expression. *p*-value < 0.05. (**C**) Immunohistochemistry of tumor tissues by CHA treatment, *n* = 3. Scale bar: 100 μm. (**D**) Immunohistochemistry of tumor tissues with ACAT1 knockdown, *n* = 3. Scale bar, 100 μm. (**E**) Overexpression of TPK1 in Be(2)-M17 and SH-SY5Y cells, *n* = 3. (**F**) Knockdown of TPK1 in shACAT1 #2 stably transfected cell lines, *n* = 3. (**G**) PDH activity in Be(2)-M17 and SH-SY5Y cells with the overexpression of TPK1, *n* = 3. (**H**) A model diagram of CHA modulation of the ACAT1-TPK1-PDH pathway promotes neuroblastoma differentiation. For **C**, * *p* < 0.05, ** *p* < 0.01, *** *p* < 0.001, and **** *p* < 0.0001, compared to the control. For (**D**–**G**), * *p* < 0.05, ** *p* < 0.01, *** *p* < 0.001, and **** *p* < 0.0001, compared to the vector.

Since thiamine metabolism is involved in ACAT1-mediated neuroblastoma cell differentiation and CHA regulates PDH activity, we focused on the relationship between TPK1 and neuroblastoma cells differentiation. The PCAT database showed that neuroblastoma patients with high TPK1 expression had corresponding low ACAT1 expression, suggesting a negative correlation between TPK1 and ACAT1 (Figure 6A). Patients with high TPK1 expression had a high overall survival rate (Figure 6B). Immunohistochemical showed that the expression of TPK1 was obviously increased in the tumor tissues of CHA-treated animals compared to the control group (Figure 6C). In addition, TPK1 was highly expressed after silencing ACAT1 (Figure 6D). Next, to confirm whether TPK1 regulates the differentiation of neuroblastoma cells, Be(2)-M17 and SH-SY5Y cells overexpressed TPK1. The overexpression of TPK1 increased the level of the differentiation marker (MAP2) (Figure 6E). Conversely, the knockdown of TPK1 downregulated the expression of MAP2 in the absence of ACAT1 (Figure 6F). Functionally, PDH activity was enhanced after overexpression in both neuroblastoma cell lines (Figure 6G). These data indicate that CHA promotes neuroblastoma cell differentiation via the thiamine metabolic pathway (Figure 6H).

## 3. Discussion

Since it is a natural small-molecule compound, CHA has excellent safety and antitumor activity [18]. However, the role of CHA in neuroblastoma differentiation has not been reported. Here, we studied the potential of CHA to induce the differentiation of Be(2)-M17 and SH-SY5Y cells. A new mechanism of CHA-induced differentiation was elucidated based on the metabolic ACAT1-TPK1-PDH pathway.

Traditionally, neuroblastoma is usually treated with radiotherapy, molecular targeted therapy, or immunotherapy; however, neuroblastoma is highly heterogeneous, which limits its efficacy and leads to drug resistance. Currently, natural products are also listed as promising drug candidates due to their low toxicity. For example, isoliquiritigenin down-regulates ATP in cells to induce cell death [19]. Curcumin can induce mitochondrial dysfunction to cause cell death, and retinoic acid induces differentiation therapy in neuroblastoma [20,21]. The overexpression of c-MYC in neuroblastoma has been reported to block neuroblastoma differentiation and developmental arrest, thereby promoting the malignant phenotype of neuroblastoma [22,23]. Neuroblastoma could differentiate into mature neurons when stimulated by a good differentiation inducer. Studies have shown that neurons are terminally differentiated cells that are completely dependent on OXPHOS for their energy and that mitochondrial dysfunction causes neuronal cells to tend to die [24]. Our study examined the phenotypes of CHA-induced differentiation cells, demonstrating that differentiated cells lost the malignant potential of tumor cells, including reduced proliferation ability, cycle arrest in the G0/G1 phase, and decreased migration and invasion ability. 

Previous studies have shown that mutant ACAT1 can deacetylate PDH and inhibit tumor proliferation. Therefore, ACAT1 is regarded as a potential anticancer drug target [25,26]. Our study showed that CHA inhibits the enzymatic activity of ACAT1. However, the relationship between ACAT1 and neuroblastoma differentiation has not yet been reported. Here, we report that silencing ACAT1 promotes cell differentiation in Be(2)-M17 and SH-SY5Y cells.

Studies have shown that when stem cells are committed to differentiation, glycolytic metabolism is transferred to mitochondrial OXPHOS to meet the increased cell energy requirements of differentiated cells [27,28]. The metabolic transition from glycolytic to mitochondrial OXPHOS is becoming increasingly important in stem cell differentiation [29]. The “Warburg effect” ensured the energy needs of tumor cells while also speeding up the biosynthesis of nucleic acids, fatty acids, and phospholipids to maintain tumor cell growth [30,31]. The normal differentiation of cells mainly obtains energy via oxidative respiration (tricarboxylic acid cycling and OXPHOS). This metabolic disorder in tumor cells also provides targeted opportunities for treatment. Mitochondrial function is known as a “tumor suppressor”, and its restoration of OXPHOS has been proposed as a therapeutic target. Reagents that inhibit glycolysis have entered clinical testing in cancer patients [32,33,34]. In our study, PDH activity was significantly enhanced in both CHA-induced differentiated cells and ACAT1 knockdown cell lines, and OCR results showed that OXPHOS function was restored, suggesting that mitochondrial OXPHOS recovery may be an important factor driving neuroblastoma differentiation.

Nutrition in the tumor environment can affect the metabolism of cancer cells and has some influence on the occurrence and development of cancer [35,36]. Thiamine is a water-soluble vitamin that is essential for the activity of four key enzymes in cell metabolism, including PDH, alpha-ketoglutarate dehydrogenase, transketolase, and the branched alpha-ketolase dehydrogenase complex [37]. Thiamine is converted into the active diphosphate TPP, the only known thiamine phosphate that functions as a coenzyme factor under the action of TPK1 [38,39]. In our study, metabolomics analysis revealed that the knockdown of ACAT1 could activate the thiamine metabolic pathway, which is attributed to the upregulation of TPK1 expression, and further restore the activity of PDH and the function of OXPHOS in tumor cells. However, the molecular mechanism by which ACAT1 regulates TPK1 expression requires further investigation.

## 4. Materials and Methods

### 4.1. Ethical Approval of the Study Protocol

This study was carried out in accordance with the Code of Ethics of the World eMedical Association (Declaration of Helsinki) and with national legislation and institutional guidelines. A public database of neuroblastoma patient data analysis was approved by the Ethics Committee of Peking Union Medical College (YW2018-018-01). Animal experiments were approved by the Ethics Committee for Animal Experiments of the Institute of Materia Medica, the Chinese Academy of Medical Sciences, and Peking Union Medical College and were conducted in accordance with the Guidelines for Animal Experiments of Peking Union Medical College (00003568).

### 4.2. Reagents

The antibodies used in this study were as follows: anti-MAP2 (Abcam, Cambridge, UK, ab32454, 1:1000); anti-ACAT1 (Cell Signaling Technology, Boston, MA, USA, #44276, 1:1000); anti-NeuN (Cell Signaling Technology, Boston, MA, USA, #24307, 1:1000); anti-Ki67 (Cell Signaling Technology, Boston, MA, USA, #9449, 1:500); anti-TPK1 (proteintech, Chicago, IL, USA, 10942-1-AP, 1:1000); anti-β-actin (ZSGB-BIO, Beijing, China, TA346894). CHA was obtained from Jiujiang Biochemical Engineering Technology Development Co. (Chengdu, Sichuan, China). CHA for cell assays was dissolved in dimethyl sulfoxide (DMSO) at an appropriate concentration in cell experiments, and CHA for injection was dissolved in normal saline at the desired concentration in animal experiments.

The short hairpin RNA (shRNA) sequence against ACAT1 was purchased from IgeBio (Guangzhou, Guangdong, China). The target sequence of ACAT1-#1-specific shRNA was 5′-CGAAATGAACAGGACGCTTATCTCGAGATAAGCGTCCTGTTCATTTCG-3′ and that of ACAT1-#2-specific shRNA was 5′-GCCTTTAGTCTGGTTGTACTACTCGAGTAGTACAACCAGACTAAAGGC-3′; control (PKO.1-puro) shRNAs were synthesized by IgeBio. siRNAs targeting TPK1 were purchased from KeyGEN BioTech (Nanjing, China). All plasmids were transfected using the Lipofectamine 3000 transfection reagent, following the manufacturer’s instructions (Invitrogen, Carlsbad, CA, USA).

### 4.3. Cell Culture

Be(2)-M17 and SH-SY5Y cell lines were obtained from the Basic Research Institute of the Peking Union Medical College (Beijing, China). Be(2)-M17 cells were cultured in DMEM medium, and SH-SY5Y cells were cultured in the RPMI-1640 medium. The medium was supplemented with 10% fetal bovine serum (FBS) and an appropriate amount of penicillin/streptomycin (P/S). Cells were cultured in an incubator at 37 °C in a humidified environment with 5% CO_2_.

shACAT1-stable cell lines were constructed by lentivirus infection. Briefly, we constructed the shACAT1 plasmid, transfected the packaged lentivirus into Be(2)-M17 and SH-SY5Y cells, and then obtained stable cell lines using puromycin screening.

### 4.4. Public Databases Analysis

Data for patients with neuroblastoma were downloaded from the PCAT database “http://pedtranscriptome.org/?home (accessed on 24 April 2023)” [40]. We analyzed the correlation between TPK1 and ACAT1 in neuroblastoma patients using the PCAT database and the effect of TPK1 on the survival of neuroblastoma patients.

### 4.5. Western Blotting Assays and Immunohistochemistry

Cells were fully lysed on ice for 30 min in the RIPA buffer (High Efficiency) (Solarbo, Suzhou, China) containing a 1% protease inhibitor cocktail (TargetMol, Boston, MA, USA), and then protein content was determined using the BCA Protein Quantification Kit (Beyotime Biotechnology, Shanghai, China) and homogenized to the same concentration based on quantification results. Equal amounts of proteins were electrophoresed in SDS-polyacrylamide gel and then transferred to PVDF membranes using the wet method. The PVDF membranes were blocked in 5% skimmed milk for 1 h and then incubated with the corresponding primary antibody overnight at 4 °C. The membranes were then incubated with secondary antibodies for 1 h. Blots were visualized using a chemiluminescence detection kit (BIORAD, Hercules, CA, USA).

The tumor tissue was quickly removed and fixed in 4% paraformaldehyde overnight, and then 2 mm thick paraffin-embedded slides were prepared. The universal two-step test kit (ZSVB-BIO, PV-9000) was used for immunohistochemical testing. According to the manufacturer’s instructions, slides were dewaxed and hydrated, underwent antigen repair, blocked endogenous peroxidase, incubated the corresponding primary antibody overnight in a wet box at 4 °C, and then a reaction booster solution and secondary antibody were added. After DAB color development and hematoxylin restaining, dehydration, transparency, and sealing procedures were carried out and finally captured under a fluorescence microscope.

### 4.6. RNA Extraction and Real-Time RT-PCR

Total cellular RNA was extracted using a rapid RNA extraction kit (ES Science, Shanghai, China). After quantification, all-in-First-Strand cDNA synthesis SuperMix for qPCR (TransGen Biotech, Beijing, China) was used for cDNA reverse transcription synthesis. Finally, real-time RT-PCR was performed using SYBR Green fluorescent dye (TransGen Biotech, Beijing, China). The cycle parameters that were executed are listed as follows: initial denaturation at 95 °C for 10 min; 40 cycles of 95 °C for 15; 60 °C for 10 s; and 72 °C for 25 s.

The primer sequences involved in the study are shown in the Table 1.

### 4.7. Migration Assay

Cells treatment using CHA (5 μΜ or 25 μΜ) or ACAT1 knockdown were digested with trypsin and resuspended in a medium containing 1% BSA. Then, 100 μL of cell suspension (20,000 cells) was inoculated into the upper chamber of a Transwell 24-well plate (Corning Costar, Tewksbury, MA, USA), and the Transwell comprised a porous polycarbonate membrane (pore size of 8 μm). The lower chamber contained 700 μL of a medium supplemented with 20% FBS. After incubation for 24 h, cells were fixed using 4% paraformaldehyde and then mechanically removed from the upper surface of the filter membrane with a cotton swab. The cells were then stained with 0.1% crystalline violet solution for 15 min, washed with PBS, air-dried, and imaged under a microscope.

### 4.8. Soft Agar Cloning Assay

In this study, 1.2% agarose and 2× medium were mixed in equal proportions and then evenly dropped into a six-well plate to make the base gel. After CHA treatment for a week, the cells were collected and the same number of cells (5000) was added to the corresponding mixture of 0.7% agarose and 2× medium for each group. Then, the suspension was successively added to the six-well plate to make a upper gel, and it was placed into the cell incubator. After overnight incubation, a cell culture medium (200 μL) was added to the six-well plate avoid gel drying. Cells were cultured for two weeks and captured under a microscope.

### 4.9. Flow Cytometry Assay

EdU and cell cycle assays were performed using a Guava EasyCyte flow cytometer (Luminex, Austin, TX, USA). In the EdU assay for cell proliferation, cells were incubated with pre-formulated 10 µM EdU (Beyotime, Nanjing, China) for 2.5 h. EdU was chemically combined with a click reaction solution (Azide 594) for 30 min. For cell cycle assessment, cells were stained with propidium (PI) and analyzed by flow cytometry.

### 4.10. ACAT1 Enzyme Activity Assay

ACAT1 activity was determined in vitro according to the previous study description [26]. In total, 100 ng of purified ACAT1 protein was added into the detection system, which contained 50 mM Tris-HCl (pH 8.1), 20 mM MgCl_2_, 40 mM KCl, 10 μM AcCoA, and 60 μM CoA. The absorbance was measured at an OD of 303 nm using a BioTek spectrophotometer.

### 4.11. Mitochondrial Membrane Potential Measurement

For the detection of mitochondrial membrane potential, JC-1 (Beyotime) was used to assess changes in membrane potential. Cells were incubated in the JC-1 staining working solution for 20 min at 37 °C. Observations were carried out using a fluorescence microscope.

### 4.12. Metabonomics Analysis

The cells were collected and removed from the cell medium, washed three times in pre-cooled PBS, digested with pancreatic enzymes, collected into 1.5 mL EP tubes, centrifuged and washed again for counting, retained cell clumps after centrifuge, and then quickly placed in liquid nitrogen for 30 s and transferred to −80 °C for preservation. The follow-up metabolomics analysis was commissioned by Shanghai Biotree Biomedical technology Co., LTD (Shanghai, China).

### 4.13. Cellular Metabolism Measurements

The Seahorse Bioscience Extracellular Flux Analyzer (XF24; Seahorse Bioscience, CA, USA) was used to monitor cellular oxidative phosphorylation in real time. Briefly, 10,000 cells were inoculated in a 24-well plate designed for XF24 and supplemented with 200 µL of suitable growth medium, and the cells were cultured overnight. Prior to measurement, cells were washed once with XF DMEM base medium containing 10 mM of glucose, 1 mM of sodium pyruvate, and 2 mM of glutamine; then, 500 µL of prepared XF DMEM medium was added to each well and incubated for 1 h without CO_2_. OCR was then measured during a typical 8 min period of mixing (2–4 min), standing (2 min), and measuring (2–4 min), as recommended by Seahorse Bioscience. Baseline OCR levels were first recorded, and then OCR levels were recorded after the continuous use of related inhibitors: Oligomycin (0.5 µM), FCCP (1.0 µM), and Retenone/Antimycin A (0.5 µM).

### 4.14. Subcutaneous and Intracranial Xenograft Neuroblastoma Models

Balb/c nude mice (male, 6–8 weeks) were purchased from Beijing HFK Bioscience Co., Ltd. (Beijing, China), and kept in a pathogen-free animal facility. The mice were subcutaneously inoculated with 1 × 10^6^ Be(2)-M17 and SH-SY5Y cells. Seven days later, the mice were randomly divided into three groups (*n* = 7). The three groups received intraperitoneal injections of vehicle, 20 mg/kg CHA, and 40 mg/kg CHA once a day for 14 days. The tumor diameter was measured with a caliper every 3 days, and tumor volume was calculated as V = 1/2 × L × W^2^, where L is the maximum length and W is the maximum width of the tumor. Tumors were dissected and immobilized in formalin for immunohistochemical analysis. 

An orthotopic xenograft model of neuroblastoma was established using 5 × 10^5^ SH-SY5Y cells. In short, a 5 µL cell suspension was injected with a microinjector at the site at 2 mm to the right, 0.8 mm to the anterior bregma, and 3.5 mm below the skull in nude mice. After a three-day recovery period, the mice were randomly divided into 3 groups and injected with saline, 20 mg/kg CHA, or 40 mg/kg CHA (i.p.) once a day for 14 days. The body weights of the mice were measured daily, and MRI analysis was performed when the body weights of the mice were reduced by approximately 20%. The parameters used in the scans to optimize gray/white matter contrast were as follows: T2_TurboRARE, with TR/TE = 5000/40; six averages; a 20 × 20 field of view; and a 0.5 mm slice thickness. All brains were scanned over 14 sessions. The brains of mice were dissected and immobilized in formalin for H&E staining.

### 4.15. Statistical Analysis

All data were presented as the mean ± SD, or SEM. Data were analyzed with a *t*-test or ANOVA test. Multiple comparisons were performed and analyzed using Dunnett’s test. Significance was defined at *p* < 0.05. 

## 5. Conclusions

In summary, our study presents a new mechanism for CHA-induced neuroblastoma differentiation. CHA promotes neuroblastoma differentiation by inhibiting mitochondrial ACAT1. The abolishment of ACAT1 upregulated the expression of TPK1, activated thiamine metabolism, and restored OXPHOS in cells. This study provides a theoretical basis for the clinical treatment of neuroblastoma with CHA and provides an attractive target and scheme for the treatment of neuroblastoma.

## Figures and Tables

**Table 1:** The sequences of target gene primers.

Gene	Forward Primer	Reverse Primer
*MAP2*	CTGCTTTACAGGGTAGCACAA	TTGAGTATGGCAAACGGTCTG
*NeuN*	CCAAGCGGCTACACGTCTC	CGTCCCATTCAGCTTCTCCC
*GAPDH*	GTGGACCTGACCTGCCGTCT	GGAGGAGTGGGTGTCGCTGT

## Data Availability

Data are contained within the article or Supplementary Materials.

## Supplementary Materials

The following supporting information can be downloaded at https://www.mdpi.com/article/10.3390/ph16060877/s1. Figure S1: The abilities of migrate and invasion were decreased in the differentiated cells.; Figure S2: CHA reduced tumor burden in both subcutaneous and orthotopic xenograft models; Figure S3: CHA inhibited ACAT1 enzyme activity.
