# Supplementary material for: Chlorogenic Acid Induced Neuroblastoma Cells Differentiation via the ACAT1-TPK1-PDH Pathway
Source: Pharmaceuticals (Basel). 2023 Jun 14;16(6):877. doi: 10.3390/ph16060877 (PMC10304613; doi:10.3390/ph16060877)
Supplement: Supplementary file 1 [file pharmaceuticals-16-00877-s001.zip › pharmaceuticals-2400977-supplementary.pdf]

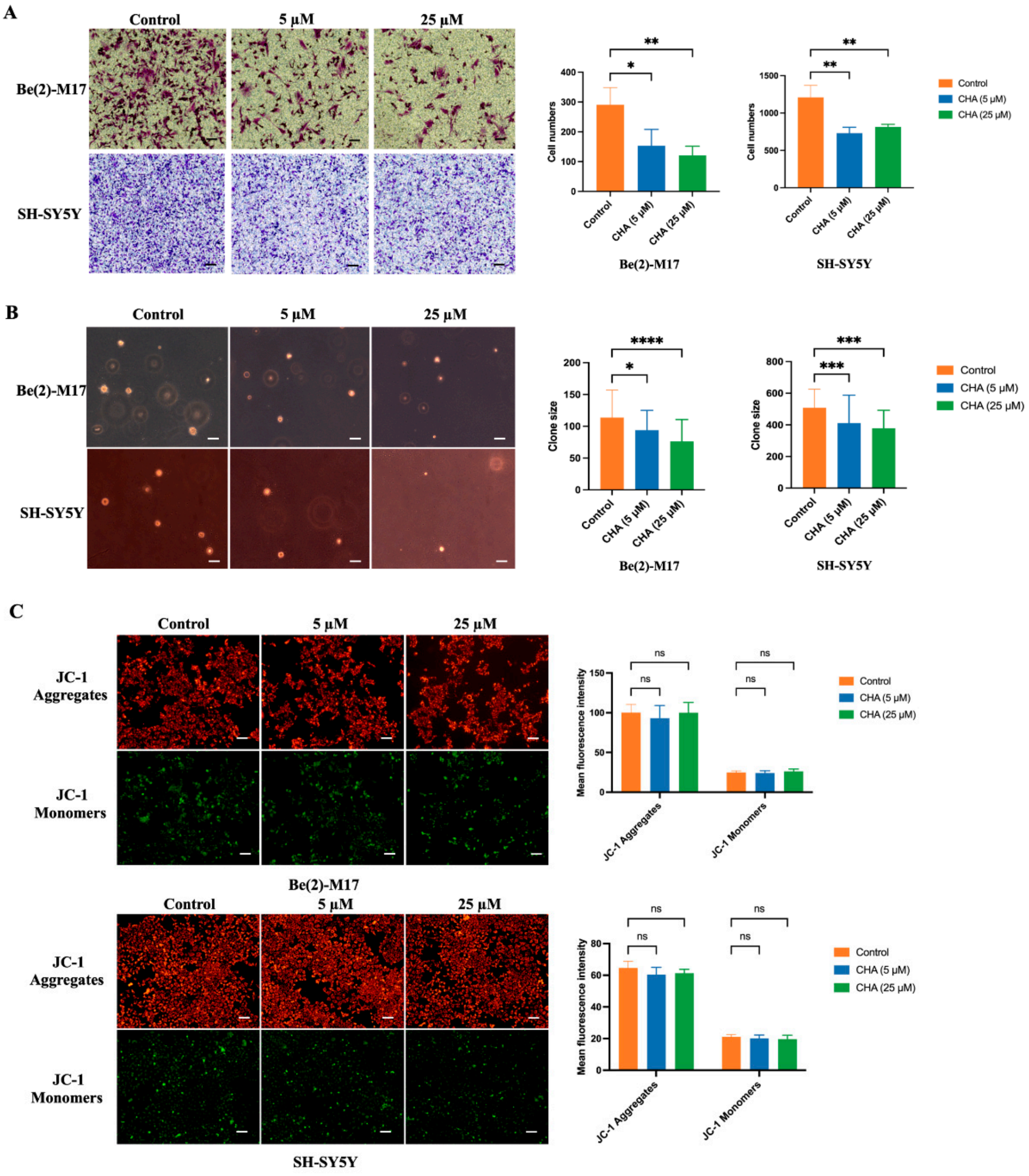


**Figure S1.** The abilities of migrate and invasion were decreased in the differentiated cells. (A) CHA inhibited the migration of Be(2)-M17 and SH-SY5Y cells, *n*=3. Scale bar, 100 μm. (B) CHA inhibited invasion of Be(2)-M17 and SH-SY5Y cells, *n*=3. Scale bar, 100 μm. (C) The mitochondrial membrane potential of Be(2)-M17 and SH-SY5Y cells with CHA treatment was observed using fluorescence microscopy. The membrane potential was determined by JC-1 staining, *n*=3. Scale bar, 100 μm. For (A-C), * $p < 0.05$, ** $p < 0.01$, *** $p < 0.001$, **** $p < 0.0001$ compared with the control, ns represents that there is no significant difference compared to the control.

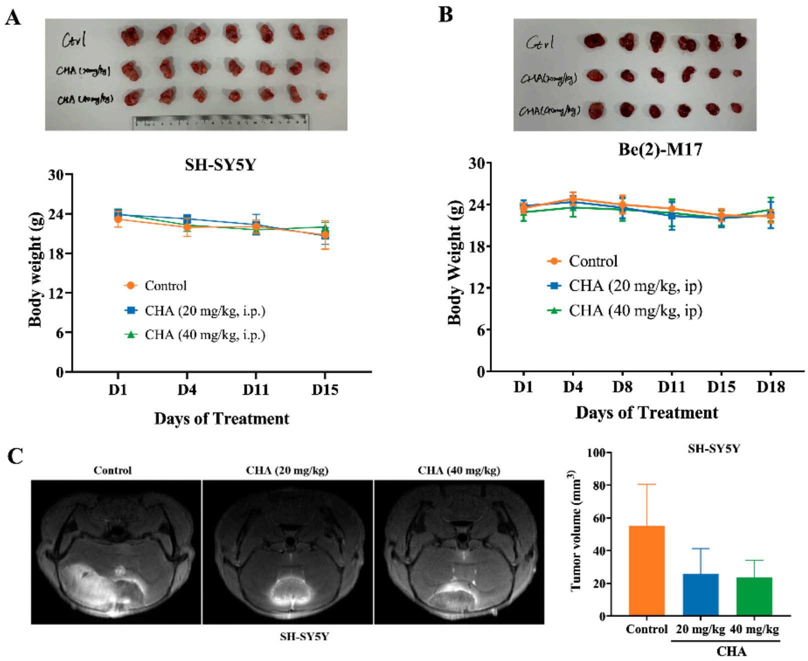


**Figure S2.** CHA reduced tumor burden in both subcutaneous and orthotopic xenograft models. (A-B) CHA could reduce tumor sizes in SH-SY5Y (*n*=7) and Be(2)-M17 (*n*=6) models and did not decrease body weight. (C) Tumor volume observation using animal MRI. The animals were inoculated with SH-SY5Y cells for 12 days, and the tumor volume was observed by animal MRI imaging. RadiAntViewer software was used to quantify tumor size, *n*=5.

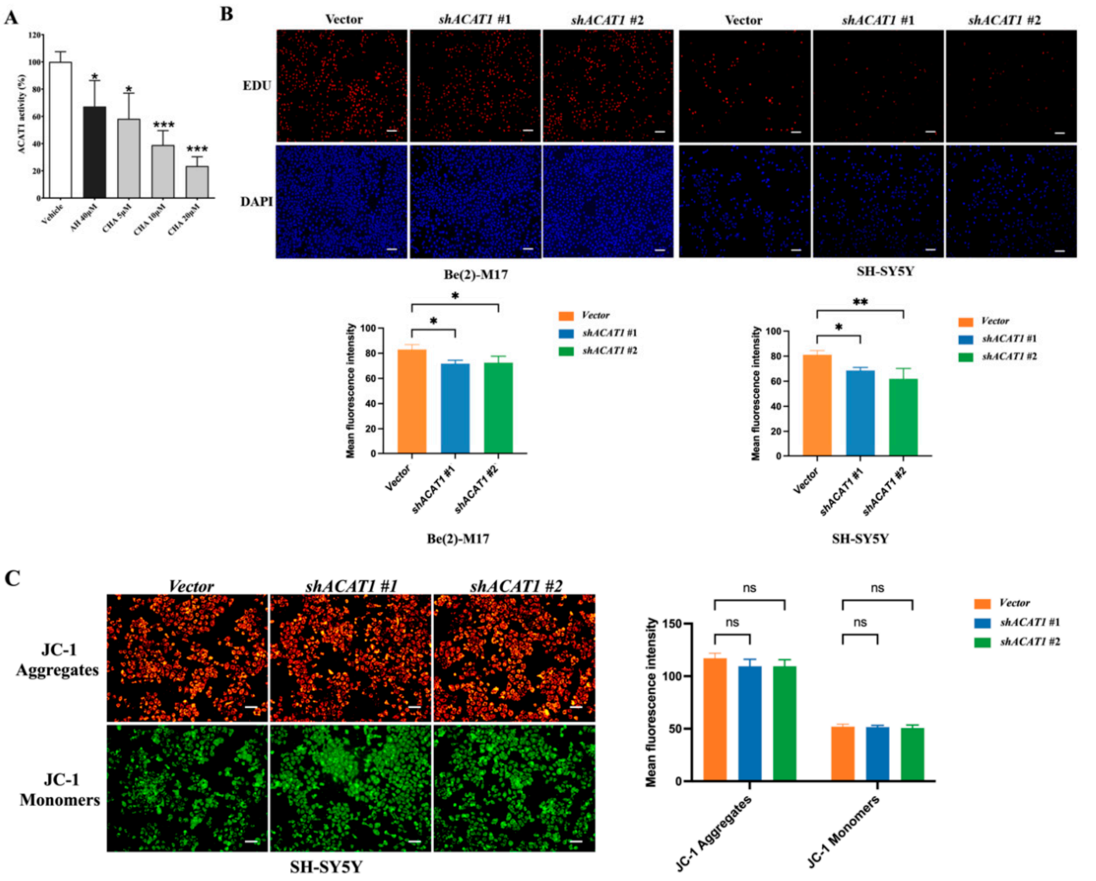


**Figure S3.** CHA inhibited ACAT1 enzyme activity. (A) CHA inhibited the enzyme activity of the recombinant protein ACAT1. (B) The mitochondrial membrane potential of the SH-SY5Y cell after ACAT1 knockdown. The membrane potential was determined by JC-1 staining, *n*=3. Scale bar, 100 µm. (C) Cell staining by Edu was detected in Be(2)-M17 and SH-SY5Y with shACAT1, *n*=3. Scale bar was 200 µm. * represents a significant difference compared with vector. For A, * $p < 0.05$, *** $p < 0.001$, compared with the vehicle. For (B-C), * $p < 0.05$, ** $p < 0.01$, *** $p < 0.001$, **** $p < 0.0001$ compared with the vector, ns represents that there is no significant difference compared to the vector.
